# 41.2 WHAT DOES EPIDEMIOLOGICAL DATA TELL US ABOUT CLOZAPINE’S EFFECTIVENESS?

**DOI:** 10.1093/schbul/sby014.170

**Published:** 2018-04-01

**Authors:** Jari Tiihonen

**Affiliations:** Karolinska Institutet

## Abstract

**Background:**

The patients included in RCTs represent a small atypical minority of the entire patient population, as up to 80–90% of patients are excluded because of mental or physical comorbidity, suicidal or antisocial behaviour, or substance abuse. Concerning clozapine, this means that those most severely ill patients having the greatest potential benefit from clozapine treatment are excluded from RCTs. Another major limitation of RCTs is that when very important but relatively infrequent phenomena, such as suicide or death is studied, exclusion of high risk patients and insufficient statistical power prevent obtaining statistically significant findings.

**Methods:**

Observational studies can overcome these obstacles by using nation-wide electronic databases of hospitalization, mortality, and filled prescriptions. However, the main problem with these observational studies is selection bias. Although the most important covariates could be adjusted in the statistical analysis, there always remains residual confounding associated with the personal characteristics of each patient. One way to overcome this problem is to use within-individual analysis, in which each individual is his or her own control. In this approach, the exposure periods of each individual are compared with the non-exposure periods of the same individual.

**Results:**

This far, 3 large observational studies using traditional between-subject analyses have found that when compared with other oral antipsychotics, clozapine is associated with the best outcome concerning risk of re-hospitalization, and 4 large cohort studies have shown that clozapine is associated with the lowest mortality. The only cohort study this far using within-individual analyses showed that in a nation-wide cohort of 29,823 patients, clozapine was associated with the lowest risk of treatment failure (defined as psychiatric re-hospitalization, suicide attempt, discontinuation or switch to other medication, or death).

**Discussion:**

A large body of observational studies shows that clozapine has better real-world effectiveness than any other oral antipsychotic treatment.

